# Advances in the production of biosurfactants as green ingredients in home and personal care products

**DOI:** 10.3389/fchem.2024.1382547

**Published:** 2024-03-26

**Authors:** Makary Nasser, Malvika Sharma, Guneet Kaur

**Affiliations:** School of Engineering, University of Guelph, Guelph, ON, Canada

**Keywords:** rhamnolipid, biosurfactant, sustainability, home and personal care products, green cosmetics

## Abstract

Home and personal care industry is currently witnessing a growing demand for sustainable and eco-friendly alternatives to synthetic surfactants. This increase is fueled by concerns over the delayed degradation and environmental impact of the latter. To this, biosurfactants possess important properties such as biodegradability, low toxicity, and renewable sourcing. These qualities position them as compelling replacements of traditional synthetic surfactants. Their diverse attributes including emulsification, antimicrobial efficacy, surface tension reduction, and foaming capability, make them well-suited choices for home and personal care products. Biosurfactants can be produced through several inexpensive and renewable sources which contributes to their commercialization potential. This article discusses various microbial derived biosurfactants including rhamnolipids, sophorolipids, mannosyl-erythritol lipids, trehalolipids and lipopeptides, unraveling and comparing their distinctive roles and advantages in the home and personal care industry. It also focuses on the recent patent innovations in the production of biosurfactants which have aimed at improving their economic viability and performance attributes. Finally, the article sheds light on the challenges and future trajectories for better integration of these sustainable biosurfactants into mainstream consumer products.

## 1 Introduction

Sustainable formulations are gaining popularity in a variety of consumer products, including cosmetics and homecare (Chin et al., 2018). The demand for sustainability is prompting a transformation in the personal care industry, potentially leading to a transition from synthetic surfactants to more eco-friendly alternatives (Cervellon and Carey, 2011). Majority of currently employed synthetic surfactants exhibit slow degradation, leading to environmental and toxicological concerns (Henkel et al., 2012). These lead to an increase in the phosphate content in aquatic systems, which is released through cleaning products and detergents, thereby leading to eutrophication (Ostroumov, 2002). One of the eco-friendly alternatives is biosurfactants. Biosurfactants are naturally occurring surfactants derived from microorganisms, such as bacteria, yeast, and fungi (Santos et al., 2016). These include a diverse range of microbial-derived amphiphilic molecules including glycolipids such as rhamnolipids, sophorolipids, mannosyl-erythritol lipids, trehalolipids, and lipopeptides (Karnwal et al., 2023). As these biosurfactants are naturally produced by microorganisms, they possess mild surfactant properties, antibacterial activity, and skin compatibility that make them well-suited for cosmetics and cleaning formulations (Mulligan, 2005). Being low molecular weight compounds, a subclass of biosurfactants known as glycolipids which includes rhamnolipids (rhamnose lipids), sophorolipids (sophorose lipids), mannosyl-erythritol lipids, and trehalolipids, have the capability to lower surface tension, function as emulsifiers, and foaming agents (Shen et al., 2019). This imparts sustainability advantages over conventional synthetic surfactants, leading to their growing prevalence in shampoos, lotions, detergents, and various consumer products. However, challenges remain in establishing consistent quality, safety, scalability and cost-effectiveness with the existing ingredients (Zajic et al., 1983). This has limited their commercialization and hence applications in the home and personal care industry despite their recognized potential. Recent research has focused on advancing high-yield microbial production and extraction techniques to improve the economic viability of biosurfactants. Advancing research into microbial biosynthesis routes, separation techniques, precision formulations, and lifecycle assessments can help unlock the vast possibilities of these renewable biosurfactants for the high demand industries of home and personal care.

This review aims to analyze recent patents and research publications related to the utilization of various types of glycolipid and lipopeptide biosurfactants in home and personal care products. Through this analysis, it focusses on the current state and trajectory of different types of biosurfactants for their integration within the industry. Analysis of patented approaches, formulations, production methods, and applications will provide insights into the unique roles and advantages of these biosurfactants for enhancing the performance and sustainability of consumer products.

## 2 Methodology for patent and literature search

The Espacenet database and Google Patent search were used for relevant patents on biosurfactants in home and personal care applications using a set of keywords. For finding the research articles, Google Scholar, PubMed, Scopus, and Science Direct were used. The keywords used included “biosurfactant”, “cosmetic”, “biosurfactant”, “personal care”, “rhamnolipid”, “skin”, “surfactant”, and “shampoo”. Additional terms such as “mild”, “sustainable”, and “antimicrobial” were also used. The search was focused on the IPC class A61K8, which is used for cleaning and cosmetics. Research articles and patents which focused solely on biosurfactant production without applications to home and personal care products were excluded. The search period was restricted to patents and publications from 2008 to 2023. Results were screened by reading abstracts and full patents and/or articles to identify innovations utilizing biosurfactants as ingredients in products like lotions, detergents, cleansers, etc. If the biosurfactant production was intended for other applications such as soil bioremediation, enhanced oil recovery, food emulsifiers, etc., but not for home and personal care products, such reports and patents were excluded from the present review.

## 3 Biosurfactants in the home and personal care industry

Microbial produced biosurfactants present a more advanced alternative to synthetic surfactants in skin contact products such as cosmetics and cleaners. Glycolipids biosurfactants such as rhamnolipids offer great surface characteristics providing essential moisturizing effects which are ideal for lotions, creams, and shampoos. Likewise, lipopeptide and sophorolipid biosurfactants have been reported to contribute antimicrobial properties, thereby enhancing formulation preservation (Lourith and Kanlayavattanakul, 2009; Kanlayavattanakul and Lourith, 2010). Sophorolipids exhibit a diverse range of functions such as emulsifiers, foaming agents, solubilizers, wetting agents (Filipe et al., 2022). Additionally, their biological activities make them valuable as active ingredients in cosmetics industry (Lourith and Kanlayavattanakul, 2009). Mannosyl-erythritol lipids are known to reduce the surface tension of water and exhibit antimicrobial activity making them essential component in skincare and anti-wrinkle cosmetics (Lourith and Kanlayavattanakul, 2009). Trehalolipids, known for their foaming capabilities, find applications in the pharmaceutical and cosmetics industries (Franzetti et al., 2010; Kuyukina et al., 2015). These functionalities of biosurfactants extend their utility across different applications in hair and skin care, as well as hard surface cleaning by home care products (Table 1).

**TABLE 1:** Applications of biosurfactants in the personal care industry.

Biosurfactant	Source	Properties	Application	References
Rhamnolipids	Bacteria	Low toxicity	Cosmetics	Lourith and Kanlayavattanakul (2009)
		Biodegradable		
		Environmentally sustainable	Personal care products	
		Antimicrobial properties		
Lipopeptides	Bacteria	Detergency	Emulsification	Kanlayavattanakul and Lourith (2010); Pilz et al. (2023)
		Foaming	Wound healing products	
		Antibacterial	Skin moisturization	
		Anti-inflammatory		
Sophorolipids	Yeast	Skin application	Pharmaceutical	Filipe et al. (2022)
		Lower surface tension	Cosmetics	
		Antiaging		
Mannosyl-erythritol lipids	Yeast	Biodegradable	Wound healing products	Shen et al. (2019); Karnwal et al. (2023)
		Emulsification	Cosmetics	
		Anti-microbial		
Trehalolipids	Bacteria, Yeast	Biodegradable	Pharmaceutical	Kuyukina et al. (2015)
	Fungi	Foaming	Cosmetics	

The production and processing of biosurfactants is based on sustainable green techniques. A systematic evaluation of factors such as pH, temperature, and shelf life ensures the quality and stability of the final products. While promising functionality has been reached in small scale production, challenges exist in establishing scaled-up systems. The cost associated with established petrochemical supply chains is demanding while the value of waste into biosurfactants addresses economic and environmental pressures. The current consumer perceptions of natural compared to historical expectations of synthetic surfactants influences product adoption. Increased large scale production of biosurfactants holds the potential to enable cleaner, safer, and more effective home and personal care products. The following sections discuss such advances in biosurfactant production and applications, as analysed from scientific literature and patents.

### 3.1 Overview of research articles on biosurfactant applications in home and personal care products

Personal care products have become a necessity, ranging from items such as soap and toothpaste to products such as deodorants, skincare, and makeup. The cosmetic industry, with its varying impact on the environment and economy, is actively introduced to challenges through the development of efficient manufacturing techniques and waste reduction (Vecino et al., 2017). As consumers move towards natural ingredients in cosmetic products, industry has begun to explore alternatives that offer comparable or superior benefits to chemical-based products.

Biosurfactants have emerged as natural compounds with significant potential in cosmetic formulations, based on their biodegradability and health impact (Lourith and Kanlayavattanakul, 2009). These compounds are produced by microorganisms such as yeast, fungi, or bacteria, and classified by chemical composition and molecular weight. Commercialization efforts are growing for various types of glycolipid as well as lipopeptide biosurfactants demonstrating a growing interest in these natural alternatives in an attempt to reflect chemically synthesized surfactants (Pilz et al., 2023). Applications are found in detergency, emulsification, de-emulsification, wetting, foaming, dispersion, solubilization of hydrophobic substances, and surface modification and are summarized in Table 2. Biosurfactants also demonstrate superior biodegradability compared to petroleum-derived surfactants.

**TABLE 2:** Advantages offered by biosurfactants in home and personal care product formulations.

Characteristic	Advantages offered by biosurfactants
Less or non-toxicity	Biosurfactants have gained market attention for their lower toxicity in comparison to chemical surfactants
Emulsification	Effective emulsifiers, facilitating the mixing of oil and water phases in cosmetic formulations
Moisturizing	Promoting skin hydration and contributing to improved skincare formulations
Biodegradability	Biologically degradable, allowing to be broken down faster than synthetic counterparts while minimizing the environmental impact
Safe for human skin	Biologically compatible and easily digestible, making them suitable for use in cosmetics, medicines, and supplements
Surface tension	Create lower surface tension contributing to their efficacy in various applications, including skincare products
Economically cheap production	Can be produced at a low cost and using renewable raw materials
Detergency	Detergency function of biosurfactants makes them effective cleansers in cosmetics

Due to their biological origin, biosurfactants contain natural fatty acids and glycols allowing for better integration with skin proteins and lipids in comparison to petroleum derived chemical surfactants (Dobler et al., 2020). This imparts biosurfactants with lower allergenicity, irritation and overall toxicity in studies evaluating skin tolerance. To this, glycolipid-based surfactants such as rhamnolipids, sophorolipids, mannosyl-erythritol lipids and trehalolipids offer structural diversity and multifunctionality presenting them as great application within cosmetics industry. Because of their low molecular weight, they reduce surface tension and interfacial tension across solids, liquids and gases, as well as enhance the solubility of hydrophobic substances in water (Abbot et al., 2022). This unique functionality of the above mentioned different classes of biosurfactants and their specific applications in home and personal care industry is discussed below.

Rhamnolipids contain either one or two rhamnose molecules, bonded to as many as three hydroxy fatty acid tail ranging from 8 to 16 carbon carbons in length. The predominant fatty acid in this composition is b-hydroxydecanoic acid. Rhamnolipids, typically produced by *Pseudomonas aeruginosa*, emerge as considerably versatile biosurfactants with significant antimicrobial activities. Their compatibility with the skin and minimal irritation makes rhamnolipids a sought-after ingredient in cosmetic products targeting diverse needs, including anti-aging and skin care. Lowering the surface tension not only increases the contact angle but also diminishes the capillary force binding the two mediums such as oil and water together (Thapliyal and Negi, 2023). Rhamnolipids have been known to retain surface activity (ST 60.0%) and emulsifying activity (EL_24_) under temperatures (4°C–121 °C), making them an ideal surfactant for the cosmetic products (Zhao et al., 2018).

**FIGURE 1:**
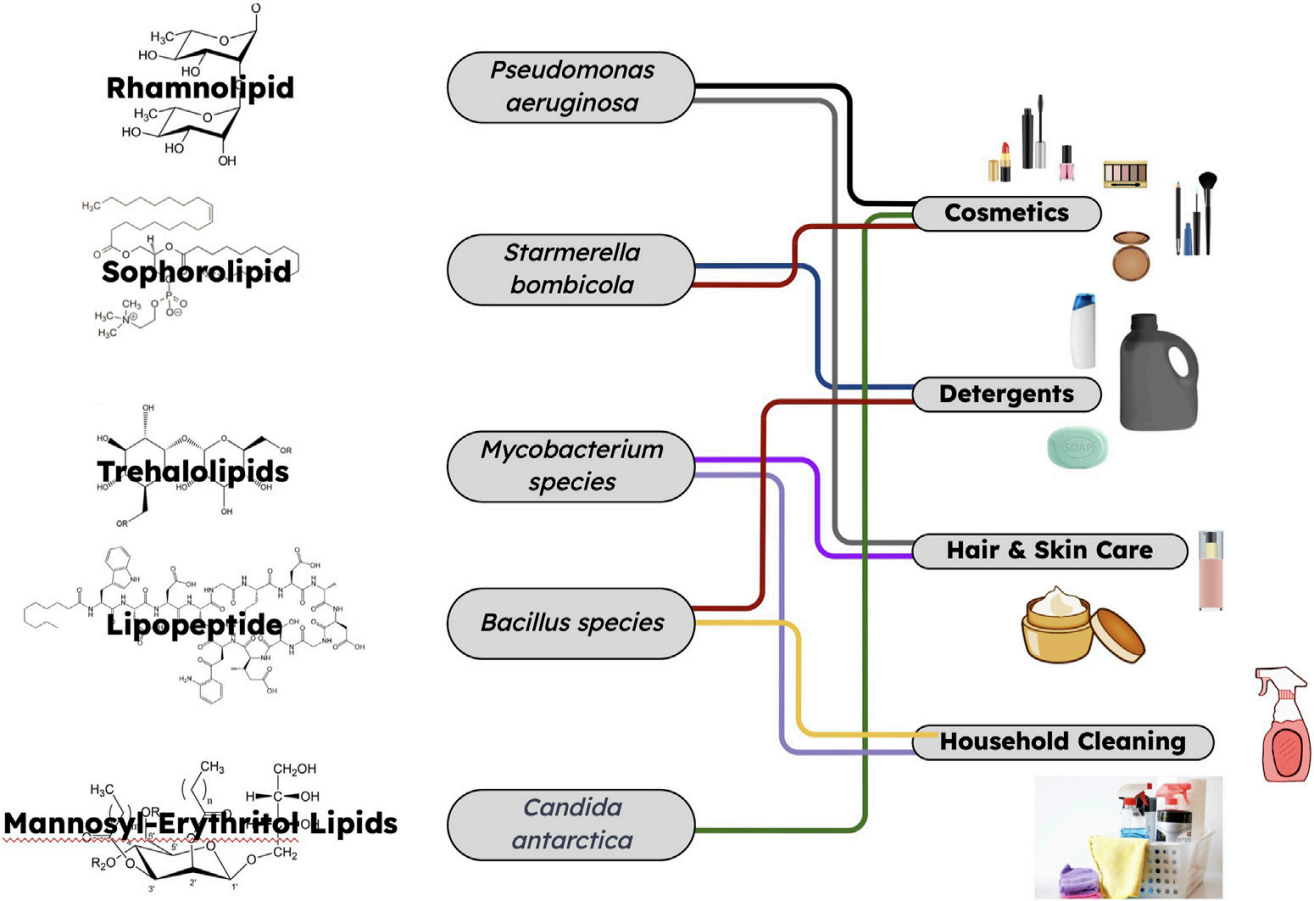
The production of natural biosurfactants by various microorganisms and their widespread applications in cosmetics, detergents, personal care, and household cleaning industries.

Sophorolipids, another kind of glycolipid biosurfactants, are produced from yeast *Candida* species, and used in cosmetics (Figure 1). With exceptional surface tension reduction and emulsifying properties, sophorolipids have become integral to various cosmetic products. It has been reported that natural sophorolipids reduced the surface tension at a water-air interface from 72 mNm^-1^ at 25°C to values ranging between 30 and 40 mN m⁻^1^ (Develter and Lauryssen, 2010). The mean droplet sizes of sophorolipid emulsions exhibit an increase as the percentage of almond oil in an almond oil-water emulsion rises for a specific duration, ranging from 500 to 5000 nm (Jiménez-Peñalver et al., 2020). Being a surfactant, its inclusion in face wash formulations was investigated by Pratap et al. (Pratap et al., 2021). These authors focused on the sustainable production of sophorolipids through utilization of waste syrup from a jaggery plant along with non-edible jaggery, corn oil and oleic acid by S*tarmerella bombicola.* Waste-derived sophorolipids showed a good foam height stability and emulsification index and were demonstrated to be used as replacements of chemically synthesized sodium lauryl sulphate in face wash formulations. Such sophorlipids-based face wash was shown to have moderate to minimal allergic reactions on all skin types including fair, medium and dark skin tones. These findings illustrate the capability of the sophorolipids to stabilize emulsions in cosmetic formulations. Sophorolipids also demonstrate skin compatibility and moisturizing benefits aligning with key consumer preferences in modern cosmetics. Such research outcomes show promise of utilization of renewable feedstock derived biosurfactants for personal care products and offer an alternative to traditional chemical surfactants.

A recent study explored biosurfactants and chitosan as natural alternatives to sodium lauryl sulfate in toothpaste formulations (Resende et al., 2019). It was discovered that biosurfactants produced by *Pseudomonas aeruginosa*, *Bacillus methylotrophicus*, and *Candida bombicola* in combination with fungal chitosan demonstrated antimicrobial activity against *Streptococcus mutans* without cytotoxicity. These biosurfactant-chitosan combinations inhibited *Streptococcus mutans* biofilm viability similarly to a commercial fluoride toothpaste. Leveraging the synergistic effects of biosurfactants and chitosan may provide a promising sustainable approach to developing effective toothpastes devoid of synthetic surfactants. While further clinical testing is still required, this demonstrates the potential for rhamnolipid biosurfactants to serve as biocompatible detergents and foaming agents in oral hygiene formulations. It also aligns with broader efforts in establishing rhamnolipids as an alternative across personal care applications based on their mild functionality and skin compatibility.

Another class of glycolipid biosurfactants, mannosyl-erythritol lipids, have gained attention for their promising properties in cosmetic applications (Lourith and Kanlayavattanakul, 2009). With a unique structure comprising a hydrophilic combination and hydrophobic fatty acids, mannosyl-erythritol lipids presents high hydrophilicity, low critical aggregation concentration, and antimicrobial activities. These characteristics position them as effective emulsifiers, dispersants, and detergents in cosmetic formulations (Figure 1). Skin care formulations incorporating mannosyl-erythritol lipids have shown efficacy in reducing wrinkles and preventing skin roughness. Additionally, the antioxidant properties of these lipids have demonstrated superior cytoprotective activity compared to arbutin which was proven by subjecting cultured human skin fibroblasts to oxidative stress induced by H_2_O_2_ (Takahashi et al., 2012). Their notable ability to lower surface tension renders them well suited for integration into cosmetic formulations. Another crucial property of mannosyl-erythritol lipids involves water retention of the epidermis and reduction in the perspiration of the skin surface inducing moisturizing property. This barrier function of the skin makes mannosyl-erythritol lipid biosurfactants potent skin care ingredients which was demonstrated in a study by Morita et al. (Morita et al., 2013). Furthermore, these authors also showed an increase in the tensile strength of the hair with the application of mannosyl-erythritol lipids based product, while maintaining the average friction coefficient, suggesting its significance as a key element in hair care products. Another research evaluated the effects of various mannosyl-erythritol lipid derivatives on skin using both a cultured human skin model and *in vivo* human studies (Yamamoto et al., 2012). It was found that these derivatives efficiently restored cell viability in sodium dodecyl sulfate-damaged skin cells, with recovery rates exceeding 80%, which was similar to natural ceramide. Notably, mannosyl-erythritol lipids derived from olive oil showed significantly higher recovery rates compared to soybean oil-derived ones. Additionally, these demonstrated promising water retention properties on human forearm skin, while increasing stratum corneum water content and suppressing perspiration. These findings suggested that mannosyl-erythritol lipid biosurfactants have strong moisturizing effects and could be valuable ingredients in skincare products, potentially for enhancing the skin barrier function.

Reports on other biosurfactants such as trehalolipids and surfactin have also shown their applications in home and personal care products. A study by Patil and Pratap (Patil and Pratap, 2018) reported a significant reduction in the surface tension of water from 72 to 24 mN m^−1^ with the application of trehalolipids. Notably, their capacity to stabilize emulsions for a minimum of 3 years makes them particularly advantageous for skincare products. A similar result for reduction of interfacial tension was also reported by Marqués et al. (Marqués et al., 2009) using trehalolipids.

Surfactin, a popular lipopeptide biosurfactant, exhibits a remarkable capability to stabilize emulsions for an extended period of 200 days. In the formulation of a cosmetic nano-emulsion with a ratio of coconut oil to surfactin at 7.4:1% w/w, the nano-emulsion remained stable after centrifugation as well as heat and NaCl treatment for 200 days (Ganesan et al., 2023). In another surfactin based product, Seweryn et al. (Seweryn et al., 2023) reported the formulation of a shower gel from surfactin produced via submerged fermentation. *Bacillus subtilis* was grown on vegetable-based raw material for 72 h and the resulting liquid was subjected to ultrafiltration and reverse osmosis to get concentrated surfactin. A surfactin rich product was formed by the addition of propanediol which was used to develop the shower gel. Incorporation of this product induced foam stability and lowered the zein value, which is a measure of severity of skin irritation. This ability to lower the zein value indicated the property of the surfactin to cause no skin irritation when included in skin care products.

Overall, rhamnolipids, sophorolipids, and other microbial surfactants effectively lower surface and interfacial tension between water and oil phases, enabling key formulation functionalities such as emulsification, foaming, solubilization and cleansing thus combining both performance and biocompatibility when used in home and personal care products. However, certain limitations exist around the production economics, yields, and purity. Large scale biosurfactant production remains more expensive than established synthetic alternatives, which limits their adoption despite their benefits. The downstream processing of diluted fermentation broths containing biosurfactants requires a prolonged multi-step purification to obtain its highest purity desirable for sensitive cosmetic applications. Natural microbial strains used for biosurfactant biosynthesis often demonstrate very low volumetric productivity. While metabolic engineering approaches are being used to boost outputs, further advances in producer strains would support the development of a more feasible commercial-scale production. On going research in these areas is expected to advance the production systems while evaluating ecological impacts and exploring novel performance attributes would establish biosurfactants in home and personal care industry.

### 3.2 Overview of patents on biosurfactant applications in home and personal care products

Recent patents have demonstrated an increasing exploration into biosurfactant applications for home and laundry cleaning formulations as a method to replace traditional synthetic surfactants. As an ever-growing emphasis on sustainability and environmental stewardship advances, the patented innovations in personal care, home, and laundry cleaning formulations underscore the industry’s commitment to greener alternatives.

Numerous innovations have been introduced, presenting various biosurfactant compositions designed for cleaning products. Desanto (Desanto, 2008) reported rhamnolipid-based formulations for household, industrial, medical, animal, and personal care applications. While rhamnolipids have shown promise as natural alternatives to synthetic surfactants, high production costs and quality have limited its widespread adoption. The inventor aimed to demonstrate efficacy of partially purified rhamnolipid blends to further bring down such sourcing expenses. Specifically for home care, a pet spray and hard surface sanitizer was developed leveraging the antimicrobial properties of sophorolipids. This patent highlights the technical and economic barriers requiring attention prior to biosurfactants such as rhamnolipids can penetrate the cleaning market. Recent research directions focused on optimizing targeted biosynthesis and biorefinery processes indicate the possibility of creating the next-generation green chemistry formulations competitive with conventional ingredients with regard to both sustainability and performance. A formulation for detergents was introduced featuring glycolipid biosurfactants, including rhamnolipids with specific polyoxyalkylene carboxylates sourced from vegetable oils (Richli, 2019). Compared to standard surfactants, these formulations demonstrated unexpected synergistic improvements in removing stains. The micelles demonstrated efficient soil solubilization, presenting advantages such as biodegradability, differing foaming capacity across pH ranges, preservative-free options, and reduced irritation potential in comparison to existing mixtures. The patent addressed environmental concerns associated with the dependance on palm oil in laundry surfactants and highlighted the toxicity risks associated with common additives. By offering effective cleaners devoid of these chemicals, the disclosed synergistic pairings not only address sustainability concerns but also meet the performance needs of consumers. This approach extends its impact to personal care applications, showcasing a transformative strategy in green chemical formulations that consider life cycle factors comprehensively. Another patent introduced a composition comprising rhamnolipids and designed for enhanced skincare and cleansing applications (Schilling et al., 2018). This formulation was developed through genetically modified *Pseudomonas putida* which overexpressed the rhamnolipid synthesis genes *rhlA*, *rhlB* or *rhlC*. Its mild physiologically compatible nature confirmed by red blood cell test outcomes ensured that the blend composition was gentle on the skin. The rhamnolipid-based composition was distinguished by its ability to leave the skin feeling soft, smooth, and refreshed after cleansing, with a notable effect that enhances the skin’s texture without an oily residue.

Considering that the penetration into home and personal care industry requires the biosurfactant production to be economically favorable and comparable to the synthetic surfactants, an invention addressed the techno-economics of biosurfactant production for their applications across various industries. Employing vegetable oils as a carbon source in biosurfactant production can incur high costs. Utilizing waste greases as cheap raw material and optimizing fermentation and extraction processes can play a vital role in reducing cost and boosting yields. Thus, Qin and Guoliang (Qin and Guoliang, 2012) optimized their rhamnolipid production process using waste greases to reduce raw material expenses by 25%–40% compared to conventional vegetable oil based fermentations. Through the combination of metabolically engineered *Pseudomonas aeruginosa* with defoaming methods, high titers up to 50 g/L were achieved. The company presented applications across agriculture, waste treatment, and pest control describing an improved affordability and scalability of this biosurfactant platform. Rhamnolipid solutions inhibited fungal phytopathogens and insect pests at concentrations ranging from 20–500 mg/L. As a supplementary agent, they improved pesticide and fertilizer performance while aiding sludge dewatering. This innovation emphasizes the versatility of tailored biosurfactants for enabling sustainable formulations for various types of consumer products. While further lifecycle and safety assessments are justified, the cross-industry relevance highlights its potential for scaled green chemical manufacturing to transform consumer product landscapes. Ultimately, solving key economic and technological challenges will be critical to unlocking the disruptive capacity of rhamnolipids as petrochemical alternatives.

In the pursuit of sustainable and effective cleaning formulations, a recently developed patent by Schilling et al. (Schilling et al., 2021a) introduced a composition designed to enhance the stability and performance of cleaning enzymes. This patented formulation incorporated a peptidase and one or two biosurfactants, with a specific emphasis on rhamnolipids and sophorolipids. The composition addressed the inherent challenge of maintaining peptidase stability in anionic surfactant systems commonly utilized in laundry and cleaning products. Traditional methods currently involve protein engineering or the use of milder surfactants. The key disclosure is the unexpected improvement in peptidase stability in the presence of anionic biosurfactants, reducing or potentially eliminating the need for protease inhibitors in formulations. The use of biosurfactants provides a sustainable solution to enzyme stability challenges. This was also demonstrated in another patent that provided a composition which combined enzymatic prowess of peptidases, specifically metalloproteases with the natural cleaning action of rhamnolipids and sophorolipids alongside anionic surfactants and water (Schilling et al., 2021b). This addressed the challenge of stabilizing peptidases within anionic surfactant systems required for long-term storage stability. The inclusion of biosurfactants improved the stability of peptidases and aided in the elimination of traditional protease inhibitors. Over 50% of the surfactants present were derived from renewable resources focusing on the commitment to sustainability. The formulation emphasized a mildness that benefited both the end-user and the environment with the potential to eliminate the use of protease inhibitors further ensuring effective cleaning in hard water conditions. Rhamnolipid-based detergent formulation were also used for fabric care in which di-rhamnolipids enhanced the foam stability and volume (Kupet et al., 2018). This formulation incorporated biodegradable surfactants and cleaning efficiencies thereby preventing fabric graying and maintaining the textile integrity. The advantages extend to its ease of formulation within aqueous systems, compatible with conventional thickeners for improved consistency, ensuring fabrics are left soft and comfortable. This presents a good example of the shift in the detergent industry towards solutions that combine high performance with low ecological footprint.

Building further upon the principles of enzymatic stability and low-irritant formulations, Urbin et al. (Urbin et al., 2013) unveiled a versatile hair and skin cleansing composition enriched with biosurfactants and fatty acids. This formulation addressed the long-lasting quest for low-irritant cleaning compositions and embraced biosurfactants, particularly rhamnolipids and sophorolipids, as primary components. The addition of oleic acid revealed favorable effects on foamability and thickening, presenting an optimized ratio of biosurfactants to fatty acids for sustainable and effective cleansing. These surfactant systems, with a “Surfactant Hydrophobe Sustainability Index” (SHSI) or a “Surfactant Sustainability Index” (SSI) greater than or equal to 0.70, signify a crucial shift towards greener detergents. By incorporating isoprenoid-based surfactants with unique branching patterns, these compositions exhibited exceptional cleaning efficiency, surpassing traditional bio-based surfactants in various performance attributes. Another patent detailed the formulations comprising water, rhamnolipids and sophorolipids, and fatty acids, with the surfactant content ranging from 1% to 30% by weight and fatty acid content from 0.1% to 20% by weight (Allef et al., 2016). This composition was designed for various personal care products, bath additives, shower gels, shampoos, and skin cleansers aiming to maximize biodegradability and skin compatibility. The incorporation of fatty acids positively affected foam and thickening properties. The invention leveraged renewable raw materials and production conditions such as fermentation to meet the growing demand for eco-friendly and skin-friendly personal care products.

Within the scope of sustainable formulations, another patent introduced a composition tailored for hair and skin cleansing, with an emphasis on human and animal body parts. Aleph et al. (Aleph and Schilling, 2017) reported a formulation comprising biosurfactants, fatty acids, and water. The versatility of this composition extended to various cleansing and care products such as shampoos, conditioners, shower gels, and body cleansing compositions. The invention addressed the problem in developing low-irritant cleaning compositions with enhanced foaming properties and storage stability, a challenge compounded by the desire for sustainable production based on renewable raw materials. The patent discussed the drawbacks of existing hypoallergenic surfactant systems, highlighting their tendency for reduced foaming ability, thereby compromising the product’s hypoallergenicity. A significant proportion of surfactants was derived from renewable sources, thereby adding further merit to this invention. As demonstrated in hand washing tests, the additional integration of oleic acid in the composition revealed favorable effects on foamability and thickening. This disclosed a composition with a carefully optimized ratio of biosurfactants to fatty acids, providing an innovative and sustainable approach to hair and skin cleansing formulations. In the process of developing sustainable and efficient cleaning solutions, another patent described inventive composition merging rhamnolipids and siloxanes in ratios ranging from 5,000,000:1 to 100:1 (Scheuermann et al., 2020). This formulation focused on environmental stewardship and significantly advancing the performance of cleaning and personal care products. Upon optimizing the weight ratio of rhamnolipid to siloxane, the patent achieved a high cleaning efficacy, enhanced odor removal, and improved experience through qualities such as increased foam production which is beneficial in products such as shaving foams.

As the above discussed patents illustrate, microbial glycolipid surfactants particularly rhamnolipids and sophorolipids can offer comparable or greater wash performance. Their functionalities like wetting, degreasing, and emulsification allow effective dirt removal under mild conditions. Properties such as low irritation make these biosurfactants well suited for skin contact. Interest has recently revolved around engineering surfactant compositions. Rather than the use of native microbial productions, advancement in bioprocessing now allows for isolation of specific surfactant compounds. Through separation techniques, this can primarily obtain specific rhamnolipid congeners, e.g., mono-rhamnolipids instead of a mixture of rhamnolipid congeners further demonstrating optimal foaming capacity. Similarly, certain biosurfactants can provide a modified functionality for more precise home care applications. While the cost and scale of production remain key considerations, the above mentioned innovations push towards the next-generation of specialized and sustainable cleaning products. Recent advances in genetic engineering of biosynthetic pathways, fermentation processes, and congener separation also continue to improve viability. Overall, glycolipid biosurfactants provide a compelling template for how biosurfactants can enable high-performing and environmentally responsible formulations. Their expanding integration presents a promising future trajectory for green chemistry innovation across the consumer product industries.

## 4 Future research and technology development

The ever growing interest and promising applications of biosurfactants, particularly glycolipids, in home and personal care products present the future research and technology development. As industry seeks to enhance sustainability, optimize formulations, and address safety and ecological concerns, many key areas have evolved for further research and development. With the discussion and evidence of the potential use of rhamnolipids as biosurfactants described above, challenges in consistent quality, safety, scalability, and cost-effectiveness continue. Future research should focus on refining and optimizing formulations for further applications within the space of cosmetic products and household cleaners. Factors to evaluate should be within the realm of pH, temperature, and shelf-life ensuring the quality and stability of future products. Innovative approaches, such as the use of waste greases as raw materials, demonstrate promising methodologies and should be further explored to reduce costs and enhance scalability. The development of environmentally optimal production systems is crucial to align with the industry’s sustainability goals. The exploration of green chemistry approaches and leveraging renewable feedstocks can contribute to the overall eco-friendliness of biosurfactant production.

While these compounds offer biocompatibility and lower toxicity compared to synthetic alternatives, further research should focus on safety assessments including long-term studies on skin tolerance, potential allergenicity, and overall toxicity to further ensure that biosurfactants meet standards for consumer use. Ecological impacts and persistence in the environment should be thoroughly investigated. Developing a life cycle assessment along with research can provide insights into the overall environmental footprint of biosurfactants, and guide formulations towards greater sustainability. Research should also explore biosurfactants in wastewater systems to assess their environmental impact beyond product use. Investigating the combination of biosurfactants with other natural ingredients to enhance antimicrobial properties can formulate effective sustainable products. This approach can lead to formulations that meet consumer preferences and contribute to the overall green chemistry movement.

Research and development should investigate into the molecular-level engineering of these compounds to understand their complex rheological behavior, shear-thinning viscosity, and other physicochemical properties. This knowledge can enable formulation tuning for specific products, such as skin creams, shampoos, and surface cleaners, based on end users’ needs. The work done in separation of designer congeners and the synthesis of tailored biosurfactants reveals new possibilities for sustainable alternatives. Further research should focus on understanding the extent of viable application possibilities of biosurfactants through their unique properties. The future of biosurfactants in home and personal care products lies primarily within the exploration of optimization strategies, safety assessments, and innovative applications.
